# Anesthetic management of a surgical patient with an acute aortic dissection complicated by hemoglobin Kansas

**DOI:** 10.1186/s40981-019-0293-3

**Published:** 2019-10-30

**Authors:** Takashi Kobayashi, Kenji Suzuki

**Affiliations:** Department of Anesthesiology, School of Medicine, Iwate Medical University, 19-1 Uchimaru, Morioka-shi, Iwate 020-8505 Japan

**Keywords:** Hemoglobin Kansas, Acute aortic dissection, Aortic replacement

## Abstract

**Background:**

Hemoglobin Kansas (Hb Kansas) is a rare disease with cyanosis.

We report a case of anesthetic management for a patient with an acute aortic dissection complicated by Hb Kansas.

**Case presentation:**

We encountered a 62-year-old male, surgical patient with an acute aortic dissection complicated by postoperative Hb Kansas. During anesthesia, his arterial oxygen saturation was low, while the partial pressure of arterial oxygen was within the normal range. The patient underwent ascending aortic replacement under hypothermic circulation arrest with a bladder temperature of 22 °C after introducing cardiopulmonary bypass. The patient was then referred to the hematology department for detailed examination and was diagnosed as having Hb Kansas through genetic analysis at 2 months after surgery.

**Conclusions:**

Except for apparent cyanosis, Hb Kansas causes no clinical problems because the delivery of oxygen to peripheral tissues may be enhanced for such patients. When we perform anesthetic management for cyanosis patients with unknown causes, it is necessary to consider the oxygen supply-demand balance.

## Background

Hemoglobin abnormalities cause cyanosis and a deviation of the partial pressure of arterial oxygen (PaO_2_) from arterial oxygen saturation (SaO_2_). Hemoglobin Kansas (Hb Kansas) is caused by a change in codon 102 AAC (asparagine) of the amino acid sequence of the hemoglobin β chain to ACC (threonine). The Hb molecule consists of two β-globin subunits and two α-globin subunits, which maintain the equilibrium between the relaxed state, with a high oxygen affinity and the tense state, with a low oxygen affinity. The binding between β102 asparagine and α 94 aspartic acid is imperative for the stabilization of the relaxed state; however, it is impossible for β102 threonine to bind to α 94 aspartic acid. The substitution pushes the equilibrium toward the tense state. Thus, patients with Hb Kansas present cyanosis; however, they are otherwise asymptomatic and do not require any specific treatment [1]. This amino acid mutation leads to decreased oxygen affinity and low heme-heme cooperativity, shifting the oxygen dissociation curve to the right compared to that for healthy people. Stamatoyannopoulos et al. reported that, in patients with low-oxygen affinity hemoglobin variants, enhanced oxygen delivery to tissues reduces the erythropoietinmediated stimulus for erythropoiesis [2]. The reduced erythropoietic drive results in a slight decrease in hemoglobin levels in hemoglobin Kansas patients. We encountered a patient who was suspected of having a hemoglobin abnormality and had to undergo emergency surgery for an acute aortic dissection. The patient was later diagnosed with Hb Kansas through genetic analysis.

## Case presentation

The patient was a 62-year-old man with a height of 167 cm and a body weight of 60 kg.

He has long shown cyanosis on his lips, and his mother had the same symptom.

The patient visited another hospital complaining of diarrhea, nausea, and dizziness, where a blood test revealed elevated liver enzymes (aspartate aminotransferase 122 IU/l, alanine aminotransferase 203 IU/l, lactate dehydrogenase 813 IU/l), total bilirubin (2.4 mg/dl), and C-reactive protein level (16.1 mg/dl), and an echocardiographic examination revealed cardiac tamponade; thus, he was referred to our hospital. Because an ascending aortic dissection (DeBakey II) was found by computed tomographic examination, the patient was advised to undergo emergency surgery on the same day of diagnosis.

He demonstrated cyanosis on his face and limbs, so even with oxygen inhalation, the percutaneous oxygen saturation (SpO_2_) was 75%. As the patient had cardiac tamponade, we suspected a decline in SpO_2_ due to heart failure. The arterial blood was venous blood-like blue-black blood. Transthoracic echocardiography revealed pericardial effusion, tricuspid regurgitation, and aortic regurgitation; however, neither ventricular collapse nor wall motion disorder was recognized, and the ejection fraction was 76%. Blood pressure was also maintained at 110–120/70–80 mmHg, and the heart rate was maintained at 70–80/min by using antihypertensive drugs, suggesting that the patient had a hemoglobin abnormality rather than a reduction in SpO_2_ due to heart failure. Therefore, the patient was suspected of having a hereditary disease. Subsequently, his case was referred to the hematology department; however, a definitive diagnosis could not be established. As the surgery had to be performed urgently, we investigated the cause of cyanosis after the surgery. Preoperative blood gas analysis and blood counts revealed the following results: PaO_2_ 219 mmHg, SaO_2_ 75%, Hb level 13.3 g/dl, hematocrit (Ht) 40.9%, white blood cell 11.30 × 10^3^/μl, red blood cell 4.24 × 10^6^/μl, and platelet 302 × 10^3^/μl.

### Anesthetic management

Anesthesia was induced with midazolam 5 mg, fentanyl 0.4 mg, and vecuronium 10 mg and maintained by the inhalation of 0.3%–0.5% isoflurane and 0.5–1.0 mg/kg/h propofol, as well as the adequate administration of vecuronium and fentanyl. After the induction of anesthesia, a pulmonary artery catheter was placed through the right internal jugular vein, and blood sampling from the pulmonary artery and radial artery was performed.

The SpO_2_ monitor showed a reading of 75%, and the blood gas analysis revealed the following results: PaO_2_ 435 mmHg, SaO_2_ 80.9%, Hb level 13.6 g/dl, and Ht 41.6% under the condition of FiO_2_ 100%. The cardiac index was 2.3 L/min/m^2^, the mixed venous oxygen saturation (SvO_2_) was 61%, and the regional oxygen saturation (rSO_2_) of the forehead was 56%. When the surgery started, the pericardiotomy was opened, and after the release of cardiac tamponade, the cardiac index increased to 3.5 L/min/m^2^ and SvO_2_ increased to 68%. However, other values did not change. The intraoperative SvO_2_ maintained the latter half of the surgery was above 60%, and the intraoperative rSO_2_ of the forehead was maintained above 50%. Furthermore, the cardiac index was maintained above 3.0 L/min/m^2^. Additionally, there was no increase in lactate.

An ascending aortic replacement surgery was performed. Cardiopulmonary bypass (CPB) was initiated by right axillary artery blood flow after the superior and inferior vena cava were removed, and ascending aortic replacement surgery was performed under hypothermic circulation arrest with a bladder temperature of 22 °C. The anesthesia time was 375 min and the operation time was 340 min. After the patient was transferred to the postoperative intensive care unit, the divergence between PaO_2_ and SpO_2_ was confirmed; however, the circulation dynamics were stable and PaO_2_ was normal. The patient underwent tracheal extubation at 15 h postoperatively. He was moved to the general ward on the 3rd hospitalization day and was discharged without complications on the 32nd hospitalization day. During hospitalization, the patient was referred to the hematology department for a detailed examination and was diagnosed as having Hb Kansas through genetic analysis at 2 months after surgery.

## Discussion

Patients with Hb Kansas do not require any specific treatment and the prognosis is good. Hb Kansas was first reported in 1961 as an Hb variant that displays a very low oxygen affinity [3]. Although there have been several reports of the disease worldwide [1], reports regarding the anesthetic management of patients with complications associated with this disease are limited. Although there are reports of general anesthesia for an infant with low oxygen affinity hemoglobinopathy [4] and sedation for a patient with Hb Kansas [5], there are no reports regarding the general anesthesia for CPB in patients with Hb Kansas.

Cyanosis includes peripheral cyanosis due to oxygen deficiency in venous blood and central cyanosis due to oxygen deficiency in arterial blood. Central cyanosis is caused by respiratory diseases and heart diseases, but there are cases of cyanosis caused by hemoglobin abnormalities. Initially, this patient was suspected to have a decline in SpO_2_ due to heart failure resulting from cardiac tamponade. However, transthoracic echocardiography revealed that his cardiac function was good, and no left-to-right shunt was observed. Moreover, his past medical history showed that he had no chronic obstructive pulmonary disease; based on the results of the blood gas analysis, we suspected that the cause of his cyanosis was a hemoglobin abnormality. However, we could not ascertain the type of hemoglobin abnormality before surgery, as we had to schedule an emergency surgery for this patient. Hypoxic hemoglobin disorder and methemoglobinemia are types of hemoglobin abnormalities. In hypoxic hemoglobin disorders, the oxygen supply-demand balance by cyanosis must be considered. In methemoglobinemia, we should pay attention to drug deterioration (nitrite, etc.). Therefore, we did not use nitrite, and oxygenation was perioperatively controlled by using PaO_2_, SvO_2_, and rSO_2_ as indicators. Additionally, because the SaO_2_ is low, the patient becomes hypoxic when there is anemia (low Hb). The arterial oxygen content (CaO_2_) after the induction of anesthesia, after cardiopulmonary bypass, and upon entering the ICU is shown below; these values were 16.0, 13.6, and 12.9 ml/dl, respectively. Although CaO_2_ was low, we did not transfuse because cardiac index, SvO_2_, and rSO_2_ were maintained and no increase in lactate was observed. If the oxygen supply-demand balance is unstable, then blood transfusion should be considered.

Except for apparent cyanosis, Hb Kansas caused no problems because the oxygen delivery efficiency of blood is rather excellent in such cases. However, there are few reports on surgery and anesthesia for patients with Hb Kansas, and the safety of surgical invasion and anesthesia in such patients is unknown. In cases of hemoglobin abnormalities, SpO_2_ is low, and this index is not effective for predicting PaO_2_; thus, an arterial blood gas analysis needs to be performed appropriately.

## Conclusion

We encountered a surgical case of acute aortic dissection complicated by postoperative Hb Kansas. When we perform anesthetic management for cyanosis patients with unknown causes, it is necessary to consider not only the pulse oximetry but also the oxygen supply-demand balance.

## Data Availability

The data in this case report are available from the corresponding author on reasonable requests.

## Abbreviations

CaO_2_: Arterial oxygen content
CI: Cardiac index
CPB: Cardiopulmonary bypass
Hb: Hemoglobin
Ht: Hematocrit
PaO_2_: Partial pressure of arterial oxygen
rSO_2_: Regional oxygen saturation
SaO_2_: Arterial oxygen saturation
SpO_2_: Percutaneous oxygen saturation
SvO_2_: Mixed venous oxygen saturation

## References

[1] Nagayama Y, Yoshida M, Kohyama T, Matsui K (2017). Hemoglobin Kansas as a rare cause of cyanosis: a case report and review of the literature. Intern Med.

[2] Stamatoyannopoulos G, Parer JT, Finch CA (1969). Physiologic implications of a hemoglobin with decreased oxygen affinity (hemoglobinSeattle). N Engl J Med.

[3] Reissmann KR, Ruth WE, Nomura T (1961). A human hemoglobin with lowered oxygen affinity and impaired heme-heme interactions. J Clin Invest.

[4] Kobayashi H, Iga S, Motohashi Y, Kumano N, Nanba K, Saeki S (2017). General anesthesia for an infant with low oxygen affinity hemoglobinopathy. Masui.

[5] Varnado-Rhodes YS (2019). Clinical cyanosis in a patient presenting for outpatient colonoscopy: a case report of hemoglobin Kansas. A&A Practice.

